# Integrating NMR and MS for Improved Metabolomic Analysis: From Methodologies to Applications

**DOI:** 10.3390/molecules30122624

**Published:** 2025-06-17

**Authors:** Patricia Homobono Brito de Moura, Guillaume Leleu, Grégory Da Costa, Guillaume Marti, Pierre Pétriacq, Josep Valls Fonayet, Tristan Richard

**Affiliations:** 1 Bordeaux INP, INRAE, Bordeaux Sciences Agro, OENO, UMR 1366, ISVV, University of Bordeaux, 33140 Villenave d’Ornon, France; patricia.homobono-brito-de-moura@inrae.fr (P.H.B.d.M.); guillaume.leleu@u-bordeaux.fr (G.L.); gregory.da-costa@u-bordeaux.fr (G.D.C.); josep.valls-fonayet@u-bordeaux.fr (J.V.F.); 2 Bordeaux Metabolome, MetaboHUB, PHENOME-EMPHASIS, 33140 Villenave d’Ornon, France; pierre.petriacq@inrae.fr; 3 UMR 1332 Fruit Biology and Pathology, INRAE, University of Bordeaux, 33882 Villenave d’Ornon, France; 4 Metatoul-AgromiX Platform, Laboratoire de Recherche en Sciences Végétales (UMR 5546), Université deToulouse, CNRS, INP, 31320 Auzeville-Tolosane, France; guillaume.marti@univ-tlse3.fr; 5 MetaboHUB-MetaToul, National Infrastructure of Metabolomics and Fluxomics, 31077 Toulouse, France

**Keywords:** metabolomics, data fusion, nuclear magnetic resonance (NMR), mass spectrometry (MS), multi-omics

## Abstract

Metabolomics, the comprehensive analysis of low-molecular-weight metabolites (typically below 1500 DA) in biological systems, relies heavily on mass spectrometry (MS) and nuclear magnetic resonance (NMR) spectroscopy. Each technique has inherent strengths and weaknesses. MS offers high sensitivity and is commonly coupled with chromatography to analyze complex matrices, yet it is destructive, has limited reproducibility, and provides limited structural information. NMR, while less sensitive, is non-destructive and enables structural elucidation and precise quantification. Recent studies increasingly employ data fusion (DF) strategies to combine the complementary information from NMR and MS, aiming to enhance metabolomic analyses. This review summarizes DF methodologies using NMR and MS data in metabolomics studies over the past decade. A comprehensive search of SciFinder, Scopus, and Clarivate Web of Science databases was conducted to analyze fusion techniques, methods, and statistical models. The review emphasizes the growing importance of DF in metabolomics, showing its capacity to provide a more comprehensive view of biochemical processes across diverse biological systems, including clinical, plant, and food matrices.

## 1. Introduction

Metabolomics, a branch of the omics sciences, focuses on the comprehensive characterization of the metabolome, defined as the complete set of low-molecular-weight metabolites within a given biological system [1]. Since its initial description by Nicholson et al. [2], metabolomics has been widely employed across various scientific disciplines in the post-genomic era. While its applications are most frequently associated with human health [3,4,5], the approach has also proven valuable in studies involving animals, microorganisms, algae and plants, environment [6], and food systems [7], thereby offering tangible benefits to medicine, agriculture, and the food industry [8].

Among the analytical platforms most commonly used in metabolomics are mass spectrometry (MS) and nuclear magnetic resonance (NMR), both of which offer distinct advantages that enhance the robustness of the results [9,10,11,12]. Each technique, however, presents inherent limitations. MS is a highly sensitive method, particularly popular in food science applications [13]. It is typically coupled with liquid or gas chromatography to enable the analysis of complex matrices, facilitating the detection and quantification of trace metabolites in biological and environmental samples, food products, and biofluids [14]. Nonetheless, MS is a destructive technique which depends on the ionization properties of compounds and does not permit complete structural elucidation. In contrast, NMR exhibits lower sensitivity than MS [15] and is generally limited to quantifying the most abundant metabolites in a sample. However, it is non-destructive and provides valuable information for structural elucidation and accurate metabolite quantification [16].

Recent advances in metabolomics have increasingly combined NMR- and MS-based approaches to harness their complementary strengths. For instance, Ghafar et al. applied UHPLC-MS and ^1^H-NMR to profile *Phyllantus acidus* leaf extracts, identifying key metabolites associated with antioxidant, α-glucosidase, and nitric oxide inhibitory activities [17]. Similarly, Razali et al. used these same techniques to explore the entomological origin of stingless bee honeys, successfully identifying species-specific markers and achieving high levels of classification accuracy [18]. Although both studies demonstrated the analytical power of each platform, the datasets were processed and evaluated separately, thereby maintaining the predictive value of each platform on its own [10]. More recently, research efforts have focused on employing data fusion (DF) strategies to construct more robust and informative models by integrating results from independently acquired metabolomics datasets [19]. DF has emerged as a growing trend, particularly as modern laboratories increasingly operate as multi-platform environments capable of investigating complex samples through a variety of analytical techniques.

In the light of this shift toward integratives approaches, increasing attention has been devoted to data fusion strategies that combine NMR and MS in metabolomics. These methodologies offer a more holistic view of biochemical profiles and have contributed to enhaced interpretation of complex biological systems. To capture the current state of this envolving field, this review aims to summarize publications from the past ten years that utilize DF strategies integrating these analytical techiniques in metabolomics-based studies. For this purpose, several databases, namely SciFinder, Scopus, and Clarivate Web of Science, were consulted to collect detailed information on the fusion strategies, analytical methodologies, and statistical models applied across these studies.

## 2. Data Fusion in Metabolomics

**Figure 1:**
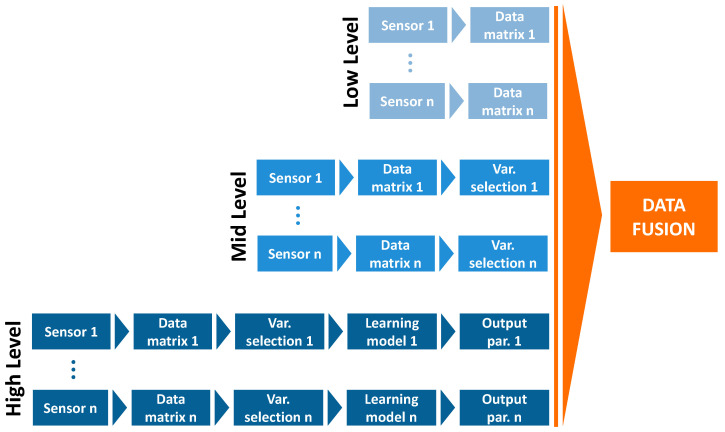
Flow chart of multi-sensor data fusion.

DF is a multidisciplinary field that allows the integration of different datasets obtained using various independent techniques to provide better insights than each approach alone [20]. In analytical chemistry, and more specifically within the field of metabolomics, the most widely used classification for data fusion is based on levels of abstraction, termed low-, mid-, and high-level data fusion, as illustrated in Figure 1 and supported by recent publications [10,21,22,23,24]. These levels represent a progression in data handling complexity: from the direct concatenation of raw or pre-processed matrices (low-level), to the integration of extracted features (mid-level), and finally to the combination of model outputs (high-level) [25]. This hierarchical representation reflects the balance between level of detail, interpretability, and computational effort. The following sections provide a detailed overview of each strategy, including their strengths, limitations, and suitability for metabolomics applications. The specific characteristics, advantages, and limitations of these approaches are detailed in the following sections.

### 2.1. Low-Level DF

Low-level data fusion (LLDF), also referred to as block concatenation [26], represents the most straightforward strategy for integrating data from different sensors. This approach enables the concatenation of two or more data matrices originating from different sources [21,23]. LLDF may be applied to raw data or to data that have undergone initial pre-processing steps [23]. In this regard, Campos & Reis detailed that this pre-processing can be divided into three stages [27]: (1) pre-processing the data by correcting the artefacts due to the signal acquisition for each sensor; (2) equalizing the contributions of each set of data collected from the different analytical sources (block) using methods such as mean centring or unit variance scaling; (3) correcting the weights of each block from the different analytical sources. Concatenation analysis tends to relate more to the block with the most significant (co)variance if not correctly handled.

In this context, the first two steps are related to intra-block scaling, while the third focuses on inter-block equalization. Forshed et al. also discussed many different intra- and inter-block scaling strategies in a concatenated approach of ^1^H-NMR and LC-MS datasets [26]. According to the cross-validation results, the authors reported that Pareto scaling ($\frac{1}{\surd \sigma^{2}}$) was the most suitable for intra-block normalization, while inter-block normalization was performed by adjusting weights to provide equal sums of standard deviation ($\frac{1}{{\left(\sum \sigma \right)}_{block}}$). However, this is not a general point, as the suitability of a scaling or normalization method depends on the sctructures and variability of each dataset. Tools like score plots, explained variance, and distance to the model can serve as objective criteria to evaluate whether the applied pre-processing improves model performance and preserves the intrinsic structure of the data.

LLDF can be explored using both supervised and unsupervised methods. From this perspective, unsupervised approaches, such as Principal Component Analysis (PCA), aim to identify common and unique patterns across datasets without relying on prior outcome information. Conversely, supervised techniques, such as Partial Least Squares regression (PLS), seek to maximize covariance within the fused matrix while integrating sample class information. Nevertheless, single-block chemometric methods like PLS or PCA are often insufficient to address the complexities of concatenation analysis. As a result, advanced methods have been developed to better integrate heterogeneous data sources. Mishra et al. provided an extensive review of sequential multiblock strategies, in the context of LLDF, applied in both exploratory and predictive contexts [28].

### 2.2. Mid-Level DF

Smolinska et al. highlight that a drawback of low-level data fusion is related to the number of observations [23], which is often much smaller than the number of variables. A highly effective way to overcome this adversity is through dimensionality reduction of the matrices separately, and this is precisely the aim of so-called mid-level data fusion (MLDF). It can be described as a two-step methodology, which (1) aims to extract the most important characteristics from the considered matrices and then (2) concatenates the outputs to build a single matrix to be processed [21,22].

Among the possible techniques for reducing matrix dimensions, PCA seems to be the most popular, with a view to subsequent concatenation to obtain a merged model. However, there are certain specificities for this strategy to be feasible: it is usually applied to first-order data [22]. For second-order data, other methods need to be employed. When they can be arranged in a data cube, the most well-known factorization method is parallel factor analysis (PARAFAC), which is able to decompose matrices into trilinear components to extract scores [29,30]. Additionally, other factorization methods have been developed to address some limitations of PARAFAC, such as the presence of artefacts in the matrices or missing data estimation problems such as PARAFAC2 [31], multivariate curve resolution–alternating least squares (MCR-ALS) [32,33], or more recently, multimodal multitask matrix factorization (MMMF) [34].

### 2.3. High-Level DF

According to Azcarate et al. [21], this is the least employed data fusion approach in analytical chemistry studies, justified by its level of complexity. Briefly, while low-level data fusion can concatenate matrices and mid-level fusion merge features, high-level fusion, also known as decision-level fusion, is able to combine previously calculated models in order to improve prediction performance and reduce the uncertainty of the final combined result [35]. These values can be qualitative, as in classification models, or quantitative, as in regression models. Typical approaches include Heuristic rules, Bayesian consensus methods, and fuzzy aggregation strategies [20,35].

HLDF is particularly advantageous when integrating heterogeneous analytical platforms such as NMR and MS, which differ in dimensionality, scale, and pre-processing requirements. Rather than fusing variables directly, this approach aggregates model-level outputs using strategies like majority voting, probabilistic averaging, or supervised meta-modeling. A relevant application is the multiblock DD-SIMCA method described by Rodionova and Pomerantsev [36], in which full distances from individual models are combined into a single cumulative metric known as the Cumulative Analytical Signal (CAS). This strategy allows the preservation of interpretability and enables the contribution of each data block to be traced in the final classification. Although HLDF may introduce interpretive complexity and may not fully exploit interactions between variables from different sources, it remains a robust and flexible framework in multiblock chemometric modeling, especially in applications involving authentication, quality assurance, and the fusion of spectroscopic and sensor-based data.

### 2.4. Potential and Limitations of DF Levels in Data Integration

Building upon the conceptual distinctions described above, it becomes clear that selecting the most appropriate data fusion strategy is a critical step for maximizing the analytical value of combined datasets, such as those generated by NMR and MS platforms. Each level of DF offers distinct advantages and presents specific challenges as already discussed, particularly when applied to metabolomics studies seeking to extract complementary biochemical information from structurally diverse data sources. LLDF, while easy to implement, may lead to unbalanced contributions if block variance is not properly normalized. MLDF addresses this by reducing dimensionality prior to merging, which facilitates interpretation but depends on the suitability of the feature extraction method. HLDF, which combines outputs from separately trained models, offers flexibility for integrating heterogeneous datasets and is particularly useful in classification and authentication tasks.

Despite its potential, the use of HLDF in metabolomics studies remains limited compared to other strategies, to the best of our knowledge. This lower adoption may reflect the need for independent model validation [22], the demand for advanced statistical expertise, and the challenge of interpreting results from aggregated outputs [23]. Such inconsistencies are particularly problematic in metabolomics, where model outputs must often be interpreted in terms of underlying biochemical mechanisms or pathways. Discrepant predictions may therefore hinder biological interpretation and compromise mechanistic insight. Moreover, the use of HLDF may require the application of decision rules, such as voting or consensus strategies, particularly when the combined models provide conflicting or contradictory information [37]. Identifying and resolving such divergences is not always straightforward and demands both methodological rigor and a solid understanding of the biological context.

Table 1 summarizes the main strengths and limitations of each data fusion level, supporting the selection of the most appropriate approach for data integration.

## 3. NMR-MS Data Fusion Applied in Metabolomics Studies

Numerous studies in the literature have combined NMR and MS data, as this integrated approach offers broader coverage of the metabolome and enhances the identification of biomarkers that are specific to each technique. However, in multiplexed analytical contexts, these studies often evaluate the datasets independently, without fully integrating them into a unified model [38,39,40]. The primary aim of this review is to highlight research that has successfully merged NMR and MS data, with a particular focus on the methodological strategies employed at different levels of data fusion.

**Figure 2:**
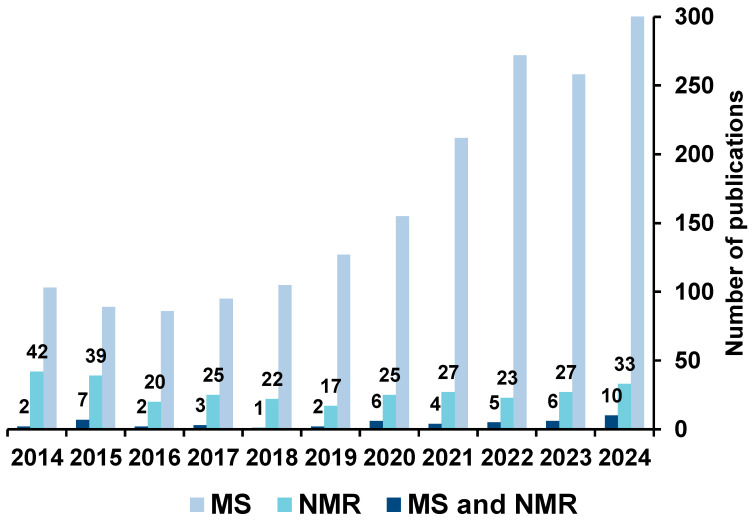
Survey of studies carried out in recent years involving NMR and MS data fusion.

In recent years, the number of studies employing data fusion strategies has grown significantly. To illustrate this trend, a search was conducted in the Scopus database using three sets of keywords: (1) (multiblock OR “data fusion”) AND MS AND NMR; (2) (multiblock OR “data fusion”) AND MS; and (3) (multiblock OR “data fusion”) AND NMR. These searches retrieved studies not only integrating MS and NMR data, but also combining these techniques with other spectroscopic outputs such as near-infrared (NIR) and ultraviolet (UV) data. The results of this literature search are presented in Figure 2, which shows that studies based solely on MS continue to rise and represent the largest share of publications. In contrast, NMR-based studies show a more stable pattern, with no significant increase over time. Meanwhile, the combined use of MS and NMR has shown an upward trend in recent years, although still with a lower absolute number of studies. This reflects a growing interest in integrative analytical strategies and highlights the perceived value of combining complementary platforms to enhance metabolome coverage and enrich biological interpretation.

Although fewer in number, studies employing multiblock analysis with NMR and MS are also showing a clear upward trend. Understanding the latest developments in this area is therefore essential for guiding future research that integrates data from these two key analytical platforms in metabolomics.

Table 2 provides an overview of NMR-MS data fusion studies published over the past ten years. The table highlights the fusion strategy adopted, the analytical techniques applied, and specific details on data processing for integration. In general, these publications exhibit a predominant focus on untargeted studies involving food matrices, plants, and clinical samples. Most of the studies utilized outputs from one-dimensional ^1^H-NMR and two-dimensional (^1^H-^1^H) *J*-resolved spectroscopy, in combination with mass spectrometry coupled to chromatographic systems such as gas chromatography (GC) and liquid chromatography (LC); one study employed direct injection (DI) mode [41].

Among the reviewed studies, LLDF was the most commonly reported strategy (18 occurrences), followed by MLDF, mentioned 6 times. As anticipated based on current trends in analytical chemistry, and to the best of our knowledge, no studies have yet applied HLDF approaches combining NMR and MS.

Given the widespread use of low-level data fusion (LLDF), it is important to examine how studies have addressed the statistical challenges associated with high dimensionality (*p* >> *n*). Table S1 summarizes the number of samples used, whether dimensionality issues were acknowledged, and the strategies applied to mitigate rank deficiency. In many cases, LLDF was implemented in settings with relatively small sample sizes (often below 100 observations), which increases the relevance of robust statistical approaches. A variety of practices were observed across the reviewed studies: while some relied primarily on general pre-processing procedures (e.g., autoscaling, UV-scaling, or block scaling), others integrated more targeted strategies to manage dimensionality. Notable examples include studies [43,50], which employed sparse or kernel-based multiblock models; [45,48,49,55], which used PCA as a dimensionality reduction step prior to supervised modeling; [56], which combined MCIA with penalized sGCCA; and [52,58], which implemented ComDim to extract global components and reduce inter-block redundancy. This range of approaches highlights the value of incorporating dimension-aware strategies to maximize model robustness in high-dimensional fusion contexts.

### 3.1. Importance of Evaluating Predictive Performance in NMR-MS Data Fusion

Evaluating the predictive performance of classification models is a fundamental step in assessing the effectiveness of NMR-MS integrative strategies. This analysis becomes particularly relevant when considering the growing complexity of multiblock datasets and the need to justify the integration of distinct analytical platforms. While many studies illustrate the benefits of combining NMR and MS, not all of them systematically report performance metrics that allow for clear comparisons between models built from individual datasets and those developed from fused data.

In this review, predictive accuracy was primarily assessed through Q^2^ values or overall classification rates. However, reporting practices varied across publications. Some studies [41,50,51] chose to focus exclusively on exploring innovative data fusion approaches, without including performance comparisons with single-block models. Other studies do provide comparisons between models built on individual datasets and those based on fusion. In some cases, however, this comparison is addressed more descriptively, for example by noting separation in coordinate score plots (such as PC1 × PC2) or by referencing permutation tests, rather than through detailed numerical metrics. While these approaches offer useful insights into data structure, they may not be sufficient for evaluating the relative robustness of the predictive models.

**Figure 3:**
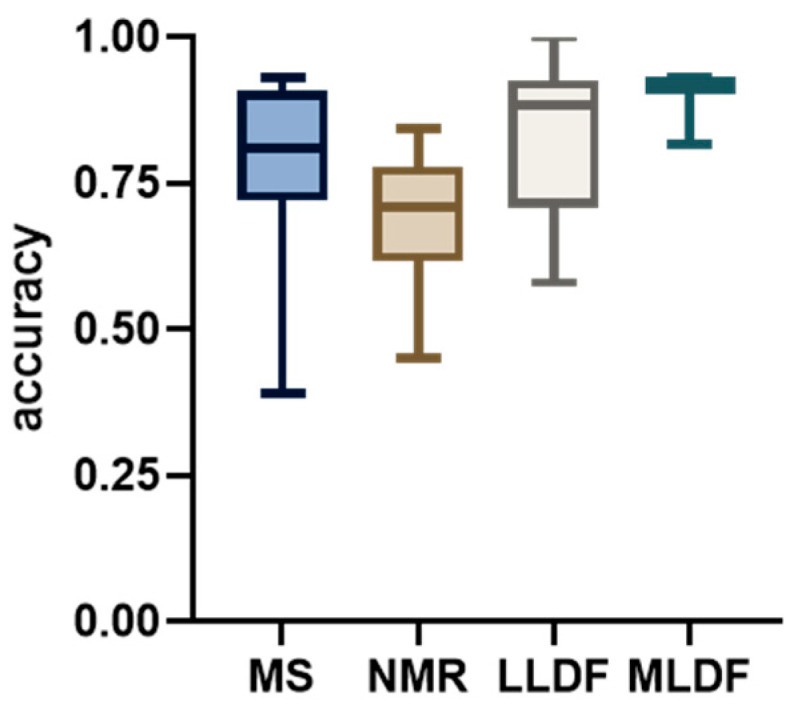
Boxplot of classification accuracy values reported in studies using MS, NMR, LLDF, and MLDF.

The box plot in Figure 3 summarizes classification results extracted from studies [41,42,49,50,51,54,56,57,58,59]. These results reflect generally strong performance, but also highlight the diversity of modeling strategies, fusion levels and validation methods used across the literature. Some papers included a direct comparison of classification performance before and after data fusion, while others chose to emphasize specific fusion strategies or biological interpretations without contrasting them with the original data blocks. Both directions are scientifically valid and contribute important perspectives. Nonetheless, when the research involves two complementary datasets, explicit comparison of their combined and individual predictive capacities provides a clearer understanding of the real benefits and potential trade-offs of data fusion.

### 3.2. NMR and MS DF in Body Fluids—Clinical Studies

Only one study on body fluids was performed using a targeted approach [42], as shown in Table 2. This study aimed to investigate metabolic differences in the urine of children diagnosed with autism spectrum disorder (ASD) compared to a healthy control group. The selection of metabolites was guided by profiles previously reported in similar populations. LLDF was applied to pre-processed datasets obtained from one-dimensional D ^1^H-NMR, 2D ^1^H-^13^C Heteronuclear Single Quantum Coherence Spectroscopy (HSQC) NMR, and LC-MS analyses, in both positive and negative ionization modes. The data matrices were concatenated and explored using PCA and OPLS-DA to uncover systematic variations among the integrated datasets. Model quality was assessed based on explained variance for predictors and responses (R^2^X and R^2^Y), as well as predictive performance (Q^2^). Statistical significance was evaluated using cross-validation analysis of variance (CV-ANOVA). This targeted strategy enabled the detection of 377 metabolites (80 from NMR and 297 from MS), among which 27 biomarkers were highlighted using variable importance in projection (VIP) scores obtained from the OPLS-DA models. These biomarkers contributed to characterizing metabolic profile alterations in Lebanese children with ASD, achieving sensitivity and specificity values above 80%.

Marshall et al. [40] conducted another noteworthy clinical study that integrated ^1^H-NMR and ESI-MS data acquired in direct injection mode (DI-ESI-MS) to investigate a neurotoxin associated with dopaminergic cell death. In this study, the authors employed the MVAPACK toolbox [62] and combined the data matrices using a multiblock approach through both supervised and unsupervised models, namely MB-PCA and MB-PLS. To ensure balanced contributions from each dataset, the blocks were scaled according to the square root of the number of variables in each matrix. For comparison, PCA and PLS were also applied separately to the individual NMR and MS datasets. All models underwent cross-validation, while only the supervised models were subjected to significance testing.

The results demonstrated that the multiblock models achieved superior separation between control and neurotoxin-treated groups compared to models based on individual datasets. These approaches also showed higher sensitivity and specificity. A particularly innovative aspect of the study was the use of backscaled loadings from MB-PLS-DA for biomarker characterization—an advanced and effective method for identifying relevant compounds. Additionally, the authors provided a detailed protocol for the combined use of ^1^H-NMR and DI-MS, including sample preparation procedures and optimization of ion source conditions to enhance metabolomic analysis.

### 3.3. NMR and MS DF in Natural Product Matrices

Regarding studies involving plant matrices, Zanatta et al. investigated the seasonal variation in the chemical composition of *Terminalia catappa* leaves [46]. Ethanolic extracts from the leaves were analyzed using two-dimensional ^1^H-^1^H J-resolved (*J*-res) NMR and UHPLC-ESI-HRMS, applying an LLDF strategy to combine the outputs. Prior to fusion, each dataset underwent intra-block scaling according to established protocols [47,48]. In this study, the fused matrix, along with the individual data matrices, was analyzed using block-wise modeling in SIMCA^®^ 17. Both supervised multivariate techniques—PLS-DA and OPLS-DA—were employed to evaluate group separations. Model validation and assessment of potential overfitting were performed via permutation tests (*n* = 100), ensuring the reliability of the classification models.

The results demonstrated that DF, when integrated with multivariate analysis, supported and complemented the insights obtained from individual datasets. Moreover, the fusion approach facilitated the identification of key metabolites associated with the observed chemical variations in *T. catappa* leaves across different environmental conditions and seasons. Notably, the study also highlighted the contribution of NMR-MS data fusion in guiding biomarker annotation. However, it is important to mention that, in the case of NMR data, the discriminant features were annotated only at the compound class level.

### 3.4. NMR and MS DF in Food Matrices

Across studies involving body fluids and natural product matrices, there is a clear predominance of supervised modeling approaches, likely due to the higher degree of similarity among the variables detected in these sample types. In contrast, publications on food matrices show a more balanced distribution between supervised [54,59,63] and unsupervised models [58,60,61]. This balanced use of supervised and unsupervised approaches in food studies may reflect a greater potential for natural clustering of samples, which is less evident in body fluids and plant matrices, where supervised models are more frequently required to achieve group discrimination.

An illustrative example is the investigation of two cold plasma processes applied to camu-camu (*Myrciaria dubia*) juice, focusing on the beverage’s flavor and aroma profiles through GC-MS and ^1^H-NMR analyses [55]. The authors justified the use of both techniques based on their complementary ability to evaluate volatile and non-volatile constituents of the juice. Following ASCII conversion, numerical matrices were generated for each dataset and exported to PLS Toolbox™ for subsequent pre-processing steps [60,64]. Then, the resulting matrices were modeled using PCA and PLS-DA, with the PLS-DA results corroborating the patterns observed in the PCA. To assess the model’s classification performance, a confusion matrix was applied. As part of the exploratory chemometric evaluation, PLS-DA was used to classify three sample groups: two corresponding to the distinct cold plasma treatments and one control group. This model explained 63% of the total variance using just two latent variables. The study confirmed that the integrated analysis yielded superior discrimination between processing methods compared to the individual analyses. Notably, the same substance-level variabilities detected in the separate datasets were also captured—and further enhanced—through the fused analysis.

A particularly relevant contribution was made by Lanza et al. [54], evaluating the discriminative capacity of fatty acid profiles (GC-MS) and polar metabolites (^1^H-NMR) in milk samples from three groups of cows fed different forage-based diets: corn silage (MS), grass–legume silage and corn (GMS), and grass hay with alfalfa (HAY). The analysis was performed using canonical Discriminant Analysis (CDA) as a supervised model, implemented in SAS software. Initially, the CDA model was trained using features from the GC-MS and NMR datasets, with the data split into training (80%) and test (20%) sets based on features with statistical significance (*p* < 0.05), using the diet groups (MS, GMS, and HAY) as prediction factors. The trained model was then applied to the LLDF matrix, and the degree of dissimilarity among forage groups was assessed through D^2^-Mahalanobis distances, using leave-one-out cross-validation. The results showed that when applied to the individual GC-MS and NMR datasets, CDA achieved only moderate discriminant capacity overall, with D^2^ values ranging from 2.6 to 31.7 (*p* < 0.001). Nevertheless, both datasets correctly separated the HAY group in the validation step. These outcomes were significantly improved through LLDF, which enhanced the model’s predictive performance, yielding higher D^2^-Mahalanobis values (7.2 to 51.2). In addition, the cross-validation step confirmed that the CDA model applied to the fused dataset was more robust and reliable, achieving greater specificity and sensitivity in authenticating milk samples from cows fed the HAY-based diet. Among the studies reviewed, this later was the only one that employed CDA as the primary discrimination strategy. Its use was associated with the advantage of generating interpretable canonical functions and clear group separation [65,66].

However, while LLDF increased robustness and discriminatory power, particularly in authenticating HAY-based milk samples, it also introduced challenges inherent to this fusion level, such as increased dimensionality and potential redundancy. These factors can complicate interpretability and increase the risk of overfitting if not properly managed. Nonetheless, the results confirmed that combining GC-MS and NMR data through LLDF provided a more reliable classification framework than either dataset alone, highlighting the promise and complexity of data fusion strategies in compositional food analysis.

Still, within the scope of food matrix studies, Jin et al. [58] explored the geographical origin of Taiping Houkui green tea using ^1^H-NMR (from polar and apolar extracts) and headspace solid-phase microextraction coupled with gas chromatography–mass spectrometry (HS-SPME-GC-MS). The authors employed both LLDF and MLDF approaches and compared them with the independent datasets using MATLAB 9.5. For LLDF, two strategies were used: (1) simple concatenation and (2) Common Dimensions (ComDim) [67]. For MLDF, two feature extraction strategies were explored: (1) variables extracted from 3D PCA loading plots and (2) the top 10 variables based on PCA scores.

In this exploratory analysis of the individual datasets, HS-SPME-GC-MS produced better clustering performance, while ^1^H-NMR (polar extracts) data yielded the least promising results. However, none of the independent techniques were able to distinguish green tea samples by origin. In contrast, ComDim-PLS loading plots from the data fusion approaches successfully identified the key metabolites responsible for differentiating the tea samples and highlighted the specific contribution of ^1^H-NMR (polar extracts) data to the overall model. These findings reinforce the idea that complementary information across datasets can yield synergistic effects. Another important aspect of the study was the application of support vector machines (SVMs) to model both LLDF and MLDF data, using linear and radial basis function (RBF) kernels. The dataset consisted of 60 samples, split into 75% training and 25% test sets. Results showed that all fusion-based models outperformed the individual datasets, with classification accuracies reaching up to 93.3%. This work presented an innovative and elegant data processing pipeline, integrating data fusion with machine learning to improve the authentication of green tea origin.

Continuing within this framework of integrating DF and machine learning, a recent study classified different vintages of the Chinese beverage Baijiu through low- and mid-level fusion of GC-MS and ^1^H-NMR datasets [57] compared with non-fused data, using Python 3.11 in the PyCharm 2020.3.2 environment and R 4.1.1 in the RStudio environment. Data processing and modeling were conducted in Python using pandas for data handling, scikit-learn for implementing machine learning algorithms and cross-validation, and matplotlib for data visualization. Meanwhile, mixOmics in R was used for multivariate analysis and for assessing variable importance via VIP scores.

**Figure 4:**
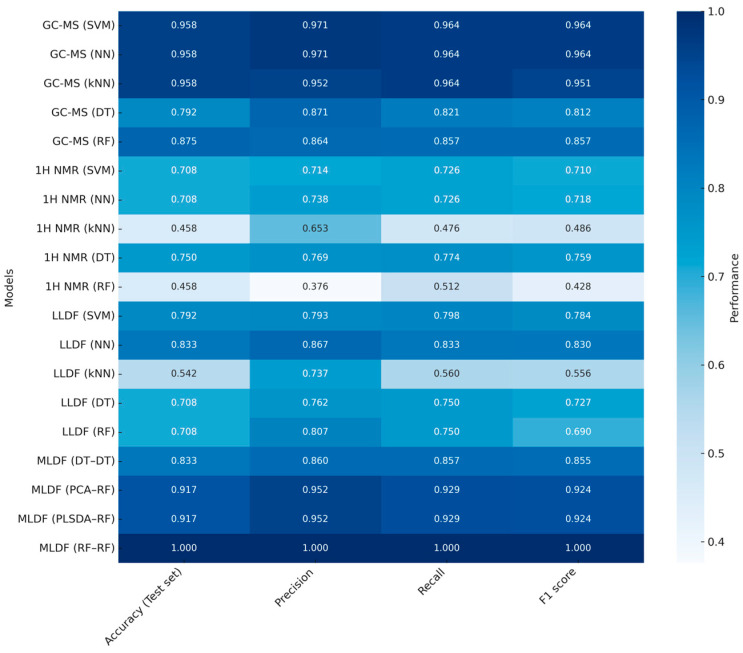
Heatmap of classification performance metrics (accuracy, precision, recall, and F1-score) for all evaluated models, based on the real results reported in the study by Chen et al. [51]. Models are labeled by analytical platform and machine learning algorithm, with mid-level fusion configurations including the dimensionality reduction method used prior to classification.

In this study, for low-level data fusion (LLDF), the datasets were directly concatenated. Subsequently, several machine learning models were applied, including support vector machines (SVMs), neural networks (NNs), k-nearest neighbors (k-NN), Decision Trees (DTs), and Random Forests (RFs). In the mid-level data fusion (MLDF) approach, dimensionality reduction was first performed using PCA, PLS-DA, DT, and RF, followed by modeling with the same set of algorithms as in the LLDF approach. In addition to fusion-based models, individual datasets (GC-MS or ^1^H NMR alone) were also evaluated using the same classification algorithms. Classification performance was assessed using four key metrics: accuracy, precision, recall, and F1-score (Figure 4).

The comparative results clearly highlight the advantage of MLDF approaches over the use of individual analytical platforms. Notably, the RF-RF configuration, in which both dimensionality reduction and final modeling were performed using Random Forests, yielded the best performance among all evaluated approaches. This model achieved perfect scores (1.000) for accuracy, precision, recall, and F1-score in both training and test sets, and recorded an out-of-bag (OOB) error rate of 0.957. Other MLDF strategies, such as PCA-RF and PLSDA-RF, also yielded high classification performance, reinforcing the chemometric advantage of combining latent variable modeling with robust classification algorithms. These elegantly constructed analyses reflect a significant trend in data fusion research: the combination of advanced machine learning tools and multi-platform analytical data to enhance the authentication and quality evaluation of complex beverages.

Among the available publications, different approaches are evident in accessing LLDF and MLDF models. Studies based on matrix concatenation typically involve different normalization strategies to equalize weights. It is also noted that there is no homogeneity in the use of software; environments such as SIMCA^®^, MATLAB, R, Python, and PLS Toolbox^TM^ are highlighted for model calculations. Despite the different approaches used, the application of data fusion in unsupervised approaches showed greater power in discriminating groups. In contrast, supervised analyses showed higher predictive efficiency than independent matrix analyses. This is possibly due to the greater metabolite coverage achieved through different analytical techniques.

Looking ahead, advances in deep learning have enabled the development of powerful in silico data integration methods for handling heterogeneous, high-dimensional omics datasets. Among these, variational autoencoders (VAEs) have been applied to integrated multi-omics analysis with promising results. For example, Hira et al. [68] proposed a deep learning framework using standard VAE and a modified version, MMD-VAE, to integrate genomic, transcriptomic, and epigenomic data from ovarian cancer patients. Their study demonstrated that these models could effectively learn compressed representations from multi-omics datasets, improving the classification of molecular subtypes and survival prediction compared to traditional dimensionality reduction techniques. Importantly, these approaches circumvent manual data scaling by learning latent representations directly from raw or minimally normalized inputs, which is particularly beneficial when integrating omics datasets with highly unbalanced feature dimensions. In a different context, Li et al. [69] introduced an adaptive data fusion strategy based on convolutional neural networks with atrous convolution to integrate multi-sensor signals for industrial fault diagnosis. Their method performed the fusion directly on multi-source inputs without requiring explicit pre-processing or manual scaling, and outperformed conventional feature-engineering approaches regarding stability and classification accuracy. Similarly, Elamin and El-Rabbany [70] applied deep convolutional neural networks to fuse UAV-based LiDAR and multispectral imagery for urban land cover classification. The joint modeling of spatial and spectral information using a U-Net with ResNet101 backbone improved classification performance relative to traditional methods, again avoiding the need for prior scaling or alignment of data channels. These studies highlight a key advantage of deep learning-based fusion models: the capacity to harmonize structurally diverse datasets, such as NMR and MS, through automated representation learning, offering a robust alternative to conventional scaling procedures often inadequate for complex metabolomics workflows.

## 4. Metabolites Covered by NMR-MS Data Fusion

In general, the annotation of compounds remains a bottleneck in metabolomics studies. The articles that reported compound annotation primarily relied on comparisons with the literature and the use of spectral repositories such as the MetLin database, Human Metabolites Database (HMDB), Biological Magnetic Resonance Databank (BMRB), Tea Metabolome Database (TMDB), Food Database, Kyoto Encyclopedia of Genes and Genomes (KEGG), and Dictionary of Natural Products (DNP). Specifically, for GC-MS data, the National Institute of Standards and Technology (NIST) library was widely employed, along with the calculation of Kovats indices.

Among the analytical tools cited, ^1^H-NMR required more resources for compound characterization. In some studies, in addition to comparison with repository data, 2D experiments (^1^H-^13^C) such as (^1^H-^1^H)-COSY, (^1^H-^13^C)-HSQC, and (^1^H-^13^C)-HMBC were described as supplementary steps in biomarker characterization.

**Figure 5:**
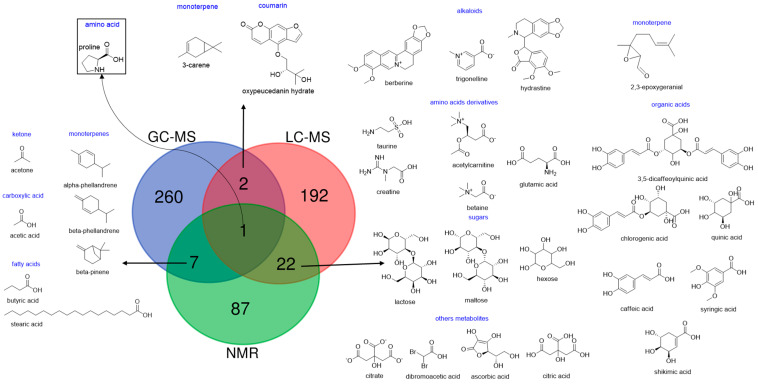
Venn diagram of the compounds covered by the NMR and MS analytical techniques, highlighting the intersections between these tools.

Figure 5 and Table S2 (Supplementary Material) illustrate the compound set coverage across the reviewed publications. The data highlight the relevance of all three analytical strategies in metabolomics studies, as each contributed to the identification of specific metabolites within clinical, plant, and food matrices. A total of 571 substances were reported. Among these, 260 (46%) were exclusive to GC-MS, 192 (34%) to LC-MS, and 87 (15%) to NMR, underscoring their distinct contributions. Only 32 (6%) compounds were detected by more than one technique, and only proline was shared across all three. This finding aligns with the previous literature, which consistently reports that only glycine and proline are commonly detected across all platforms [71]. The high number of technique-specific compounds underscores the value of data fusion approaches, which improve metabolome coverage while minimizing redundancy in data integration.

Although the analytical potential of NMR is well recognized, particularly for its ability to detect a wide range of structurally diverse metabolites without the need for derivatization or separation steps, it exhibited the lowest number of annotated compounds among the reviewed studies. This observation reflects ongoing challenges in metabolite annotation by NMR, especially when dealing with complex biological matrices. In such samples, peak overlap, signal suppression, and lower intrinsic sensitivity can hinder the resolution and assignment of individual constituents, limiting the overall identification yield.

Among the platforms most frequently employed in NMR- and MS-based DF studies, ^1^H-NMR and UHPLC-MS remain the primary analytical techniques associated with metabolomics approaches, as shown in Table 1. Both methods enable the simultaneous detection of a wide range of metabolites, particularly nitrogen-containing compounds such as alkaloids and amino acids, as well as various organic acids. This capability is crucial, as LC-MS and NMR are often complementary in the identification of known and unknown metabolites within complex mixtures.

To enhance this integrative analysis, specialized tools have been developed, including SUMMIT NMR/MS [72] and Data Fusion-based Discovery (DAFdiscovery) [73], which were developed to merge MS and NMR data. SUMMIT NMR/MS focuses on identifying chemical formulas through MS data, generating all plausible structures for each formula, and then comparing them to experimental NMR spectra to determine the most consistent molecular candidates. This workflow facilitates the structural elucidation of metabolites by integrating the strengths of both analytical platforms. DAFdiscovery, in turn, employs statistical total correlation spectroscopy (STOCSY) and statistical heterospectroscopy (SHY) to assist in metabolite annotation. These advanced tools underscore the importance of combining multiple analytical techniques to improve metabolome coverage and biomarker identification across diverse biological matrices.

## 5. Concluding Remarks

In this review, various innovative fusion strategies employing 1D and 2D NMR (^1^H, ^1^H-^1^H *J*-Res, ^1^H-^13^C HSQC) and MS (LC, GC, DI) over the past decade were considered. These strategies include classical multivariate exploratory or classificatory models, and some of their modifications, such as MB OPLS-DA, Consensus-PCA, and Sparse MBPLSR, as well as the application of machine learning tools on fused data.

In all these studies comparing results from independent matrices with merged data, data fusion techniques demonstrated a greater capacity for discrimination and classification of the considered variables. However, it is essential to validate these models, as not all accessed publications included this crucial step in establishing chemometric approaches.

The NMR-MS data fusion proves to be a powerful tool for studying complex matrices such as plants, clinical samples, food, fungi, and marine organisms. The promising data presented in publications highlight successful results in authentication and quality assessment of foods by origin, types of applied processes, and geographic region; plants regarding seasonal variations, origin, and extraction processes; and clinical studies emphasizing metabolic profile changes associated with the presence of disorders or dietary changes. These results reveal the unique metabolic coverage of each analytical technique used and their synergistic potential in biomarker discovery. Given all this, further research that involves the fusion of different analytical techniques, especially NMR and MS, is strongly encouraged.

The annotation of the observed biomarkers remains the main challenge. Nonetheless, mass spectrometry appears to offer more resources for compound characterization compared to NMR, especially in complex mixtures, even though that can be optimized through correlated NMR and MS analyses such as SUMMIT NMR/MS or Data Fusion-based Discovery.

## Figures and Tables

**Table 1:** Summary of strengths, limitations, and applications of data fusion levels.

Criterion	Low-Level	Mid-Level	High-Level
**Implementation**	Simple, direct	Feature reduction needed	Separate models required
**Data Richness**	Full information	Compressed, relevant	Outputs only
**Overfitting Risk**	High (many variables)	Moderate	Low (if models are robust)
**Interpretability**	Moderate, variable-level	Component-based	Low, indirect
**Data Compatibility**	Low (scaling required)	Moderate (pre-processing helps)	High (platform-independent)
**Application Focus**	Exploration, trend mapping	Clustering, pattern discovery	Classification, decision support
**Limitations**	Sensitive to scale; risk of bias	Loss of nuance; method-dependent	Requires expertise; low traceability

**Table 2:** Overview of published studies employing data fusion approaches combining NMR and MS analyses, categorized by biological matrix (body fluids, natural products, and food), analytical strategy, modeling techniques, and validation criteria.

Approach	Aim	Datasets	DF Level	Fusion Strategy	Stat. Modelling	Model Validation	Ref.
Body Fluid Matrices—Clinical Studies							
Targeted	Compare the metabolic profiles of patients affected by autistic disorders	LC-MS ^1^H-NMR ^1^H-^13^C (HSQC)	LLDF	Concatenation of pre-processed datasets	OPLS-DA	CV-ANOVA	[42]
Untargeted	Evaluate the effects of dietary variations on plasma responses in pigs	LC-MS ^1^H-NMR	LLDF	Concatenation of pre-processed blocks	Sparse MB-PLS	RMSECV	[43]
Untargeted	Differentiate the urinary metabolic profiles of irradiated and non-irradiated mice	LC-MS ^1^H-NMR	LLDF	Concatenation of pre-processed datasets	MB-OPLSDA	CV-ANOVA	[44]
Untargeted	Evaluate optimization methods for NMR and MS data fusion using multiblock bilinear factorization in neurotoxin analysis	DI-MS ^1^H-NMR	LLDF	Concatenation of pre-processed datasets	MB-PCA, MB-PLS	LOOCV	[41]
**Natural Products Matrices (plant, marine, and fungal sources)**							
Untargeted	Evaluate the variability in primary metabolites and aroma compounds of pines	GC-MS ^1^H-NMR	LLDF	Concatenation of pre-processed datasets	PLS-DA	Permutation test	[45]
Untargeted	Evaluate the environmental factors driving seasonal variations of a medicinal plant	LC-MS ^1^H-^1^H (*J*-resolved)	LLDF	Concatenation of pre-processed datasets	PLS-DA, OPLS-DA	Permutation test	[46]
Untargeted	Assess the influence of seasonal factors on the chemical composition of two medicinal plant species	LC-MS ^1^H-^1^H (*J*-resolved)	LLDF	Concatenation of pre-processed datasets	PLS-DA	Permutation test	[47]
Untargeted	Examine the relationships between environmental factors and the growth of an invasive weed	LC-MS ^1^H-NMR	LLDF	Concatenation of pre-processed datasets	PCA, HCA, OPLS-DA	-	[48]
Untargeted	Discriminate marine sponges	LC-MS ^1^H-NMR	LLDF	Concatenation of pre-processed datasets	Consensus PCA, MB-PLS	LOOCV	[49]
Untargeted	Classify lemon essential oils based on their extraction methods	LC-MS ^1^H-NMR	LLDF	Concatenation of pre-processed datasets	MB-PLS, consensus K-OPLS-DA	LOOCV	[50]
Untargeted	Monitor the biotransformation medium of a potential histamine H3 antagonist	LC-MS ^1^H-NMR ^1^H-^1^H (*J*-resolved)	LLDF	Concatenation of pre-processed datasets	Consensus OPLS-DA	K-fold	[51]
Untargeted	Evaluate metabolic differences of algae	GC-MS LC-MS ^1^H-NMR	LLDF	Concatenation of pre-processed datasets	MB ComDim	Permutation test	[52]
Untargeted	Determine the botanical origin and authenticity of blue cohosh	LC-MS ^1^H-NMR	MLDF	This approach analyzed the top five principal components (i.e., latent variables) of the NMR and MS datasets	PCA	-	[53]
**Food Matrices**							
Untargeted	Discriminate milk from dairy chains based on different dietary types	GC-MS ^1^H-NMR	LLDF	Concatenation of datasets	CDA	LOOCV	[54]
Untargeted	Investigate the effect of operating conditions on both non-volatile and volatile compounds in juice	GC-MS ^1^H-NMR	LLDF	Concatenation of pre-processed datasets	PCA, PLS-DA	RMSEC, RMSECV	[55]
Untargeted	Classify wines according to grape withering time and yeast strain	LC-MS ^1^H-NMR	LLDF	Concatenation of pre-processed datasets	MCIA, SPLS-DA	3-fold cross validation	[56]
Untargeted	Investigate the different vintages of Baijiu beverage	GC-MS ^1^H-NMR	LLDF	Concatenation of datasets	PCA, PLS-DA machine learning: SVM, DT, RF, k-NN	10-fold cross validation	[57]
			MLDF	Extraction of features by: PCA, PLS-DA, Decision Tree (DT), Random Forest (RF)			
Untargeted	Discriminate geographical origin of green tea	GC-MS ^1^H-NMR	LLDF	Concatenation of pre-processed datasets	PCA, ComDim-PLS	-	[58]
			MLDF	Concatenation of 3D PCA loading plots (approach 1)	PCA		
				Concatenation VIP scores (top 10-approach 2)	SVM		
Untargeted	Classify rums based on fermentation barrel, raw material, distillation method, and aging	GC-MS LC-MS ^1^H-NMR	LLDF	Concatenation of pre-processed raw data	PLS-DA	RMSECV, CV ANOVA, permutation test	[59]
			MLDF	Concatenation of features based on VIP scores			
Untargeted	Evaluate the influence of two processing methods on juice composition	LC-MS ^1^H-NMR	MLDF	Pre-processed matrices of NMR and MS were converted to ASCII	PCA	-	[60]
Untargeted	Honey origin discrimination and comparative analysis of mid-level fusion strategies	LC-MS ^1^H-NMR	MLDF	Concatenation scores of PCAs of each analytical method (no variable selection) Selection of relevant variables of each analytical method using PLS	PCA, PLS-DA	-	[61]

Nuclear magnetic resonance (NMR); Proton NMR (^1^H-NMR); Carbon-13 NMR (^13^C-NMR); ^1^H-^13^C Correlation (^1^H-^13^C); ^1^H-^1^H Correlation (^1^H-^1^H); Heteronuclear Single Quantum Coherence (HSQC); *J*-resolved spectroscopy (*J*-RES); gas chromatography (GC); liquid chromatography (LC); mass spectrometry (MS); direct injection (DI); low-level data fusion (LLDF); mid-level data fusion (MLDF); multiblock (MB); Text Export Format (ASCII). Principal Component Analysis (PCA); Partial Least Squares (PLS); Sparse Partial Least Squares (SPLS); Canonical Discriminant Analysis (CDA); Discriminant Analysis (DA); Orthogonal Partial Least Squares (OPLS); support vector machine (SVM); Random Forest (RF); Decision Tree (DT); neural network (NN); Multiple Co-Inertia Analysis (MCIA); cross-validation (CV); leave-one-out CV (LOOCV); analysis of variance (ANOVA); Root Mean Square Error of Cross-Validation (RMSECV); RMSE of Calibration (RMSEC); variable importance in projection (VIP).

## Data Availability

No new data were created or analyzed in this study.

## Supplementary Materials

The following supporting information can be downloaded at: https://www.mdpi.com/article/10.3390/molecules30122624/s1, Table S1: Overview of sample size and rank deficiency mitigation approaches in low-level data fusion studies combining NMR and MS; Table S2: Coverage of compounds in NMR-MS data fusion studies by analytical technique.
