# Supplementary material for: Integrating NMR and MS for Improved Metabolomic Analysis: From Methodologies to Applications
Source: Molecules. 2025 Jun 17;30(12):2624. doi: 10.3390/molecules30122624 (PMC12196070; doi:10.3390/molecules30122624)
Supplement: Supplementary file 1 [file molecules-30-02624-s001.zip › molecules-3693937-supplementary.pdf]

Table S1. Overview of sample size and rank deficiency mitigation approaches in low-level data fusion studies combining NMR and MS.

Ref.	Sample Size	Dimensionality issue	Pre-processing and/or strategies to mitigate rank deficiency
[42]	80	Yes	Raw concatenation of full matrices followed by UV-scaling and OPLS-DA modeling.
[43]	44	Yes	Block-level sparsity in MB-PLSR to improve interpretability and reduce overfitting
[44]	21	Yes	Permutation test and CV-ANOVA used to monitor model overfitting
[41]	Not clearly reported	Yes (likely)	Multiblock scaling using CPCA-W and MB-PLS with super-score deflation; fairness ensured by block scaling
[45]	30	Yes	PCA on each matrix after alignment (COW), baseline correction, area normalization, mean centering and block variance scaling for fair contribution in MB analysis
[46]	~39 (estimated: 13 seasonal collections — 3 replicates from 5 individuals)	Yes	Block-wise scaling using standard deviation per block; Pareto scaling; FDR filtering
[47]	24	Yes	Block-wise scaling to equalize NMR and MS contributions in multivariate models
[48]	Not specified (monthly collections over 2 years from 48 individuals per site — 2 sites; multiple plant organs)	Yes (likely)	Arcsin and log transformation of environmental variables; block-wise scaling for data fusion; dimension reduction via PCA, HCA, and supervised modeling with OPLS-DA
[49]	33	Yes	Block scaling (per-block variance normalization) prior to CPCA and MBPLS to balance variable influence and reduce dimensionality
[50]	64	Yes	Superscore-based MB-PLS-DA; Consensus OPLS-DA, block-wise normalization and weighted kernel fusion using RV coefficients to extract predictive components
[51]	42	Yes	OPLS-DA multiblock with block-wise normalization; predictive component extraction; Pareto scaling
[52]	15 (3 replicates — 5 locations)	Yes	Autoscaling; ComDim to extract global components (salience/weighting); separate modeling per block
[54]	90	Yes	Stepwise feature selection ($p < 0.05$) prior to CDA on LLDF
[55]	15	Yes	Data normalization before fusion + PCA prior to OPLS-DA
[56]	80	Yes	MCIA to project datasets into a shared latent space, followed by sGCCA (l1 penalization) for dimensionality reduction and feature selection
[57]	118	Yes	Not addressed
[58]	72	Yes	Normalization; Common Dimensions Analysis (ComDim) for extracting global components and reducing redundancy across data blocks
[59]	24	Yes	Scale normalization to reduce inter block dominance

Table S2. Coverage of compounds in NMR-MS data fusion studies by analytical technique

GC-MS	LC-MS	NMR
3-methylbutan-1-ol	digalloyl glucoside	2,3-epoxygeranial
ethyl butanoate	ethyl hydroxybutanoate-*O*-hexoside	2-hydroxybutyric acid
hex-3-en-1-ol	myricetin-*O*-hexoside	2-oxoglutarate
1-hexanol	quercetin-*O*-rhamnoside	3,5-dicaffeoylquinic acid
α-pinene	cinnamoyl glucoside	acetate
camphene	isorhamnetin-*O*-hexoside	acetic acid
β-pinene	anacardic acid (15:3)	acetone
myrcene	anacardic acid (15:2)	acetylcarnitine
α-phellandrene	anacardic acid (17:3)	adenine
limonene	anacardic acid (15:1)	alanine
γ-terpinene	anacardic acid (17:2)	alpha-ketoglutarate
terpinolene	anacardic acid (17:1)	ascorbic acid
α-fenchol	reynosin	asparagine
borneol	artabsinolide d	aspartate
4-terpineol	tartridin b	baa
α-terpineol	(12E)-9,10-dihydroxy-12-octadecenoic acid	berberine
β-caryophyllene	sitostenone	betaine
α-humulene	9-hydroperoxy-10E-octadecenoic acid	butyrate
α-bulnesene	10,16-dihydroxyhexadecanoic acid	chlorogenic acid
γ-cadinene	9,10-dihydroxystearic acid	choline
cadina-1(2),4-diene	apigenin-6-*C*-(-*O*-galloyl)-hexose	cis-aconitate
selina-3,7(11)-diene	apigenin-6-*C*-hexose (isovitexin)	citrate
α-calacorene	hydroxyursolic acid	citric acid
germacrene b	trihydroxyurs-12-en-28-oic acid (asiatic acid)	citruline
butyric acid	tetrahydroxyolean-12-en-28-oic acid	coniferin
caproic acid	luteolin-6-*C*-hexose (isoorientin)	creatine
caprylic acid	trihydroxyursadien-28-oic acid	creatinine
capric acid	3-oxo-urs-12,18-dien-28-oic acid	dibromoacetic acid
caproleic acid	magnoflorine	dimethylamine
undecylic acid	N-methylcytisine	epicathechin
lauric acid	cauloside c	epigallocatechin
lauric acid (trans)	cimifugin	ethanol
lauric acid (cis)	betaine	floridosise
tridecanoic acid	lactose	formate
myristic acid (iso)	hydrastine	formic acid
myristic acid	berberine	fructose
myristoleic acid	palmatine	fumarate
pentadecanoico acid	canadine	galactose
pentadecanoic acid (iso)	yohimbine	gamma-aminobutiric acid
14-pentadecenoic acid	corynanthine	glucose
palmitic acid (iso)	maltose	glucose-1-phosphate
palmitic acid	quinic acid	glutamate

palmitoleic acid (trans)	*O*-galloylhexose	glutamine
palmotileic acid (cis-9)	gallic acid	glycerol
margaric acid (anteiso)	di-*O*-galloylshikimic acid	glycine
margaric acid (iso)	di-*O*-galloylquinic acid	hexose
margaric acid	tri-*O*-galloylquinic acid	hippurate
heptadecenoic acid (cis-9)	(*epi*)catechin	hydrastine
stearic acid	quercetin-*O*-hexose-pentose	hydroxybenzoic acid
oleic acid (cis-9)	*O*-galloylshikimic acid	isethionic acid
elaidic acid	*O*-galloylquinic acid	isoleucine
linoleic acid	tri-*O*-galloylshikimic acid	lactate
nonadecanoic acid	tetra-*O*-galloylquinic acid	lactic acid
α-linolenic acid	coumaroylquinic acid	lactose
linolenic acid	(*epi*)catechin-(*epi*)catechin-(*epi*)catechin-(epi)catechin	leucine
rumenic acid	chlorogenic acid	malic acid
arachidic acid	quercetin-*O*-hexose-deoyhexose-deoxyhexose	maltose
eicosenoic acid	syringic acid	mannose
dihomo-gamma-linolenic acid	feruloylquinic acid	methanol
arachidonic acid	trimethyl gallate	methionine
behenic acid	quercetin-*O*-(*O*-galloyl)-hexose	methyl malonic acid
dimethyl sulfide	quercetin-*O*-hexose-deoxyhexose	N,N-dimethylglicyne
3-methyl butanal	*O*-galloyl-(*epi*)catechin-(*epi*)catechin-*O*-gallate	N-acetylcisteine
2-methyl butanal	(*epi*)catechin gallate	N-acetylglucosamine
1-penten-3-ol	quercetin-*O*-hexose	orotate
1-penten-3-one	(*epi*)catechin-(*epi*)catechin	phenylalanine
(E)-3-penten-2-one	coumaric acid	phosphocoline
1-pentanol	quercetin-*O*-deoxyhexose-pentose	phosphocreatine
(Z)-2-penten-1-ol	methyl gallate	piperine
4-methyl-3-penten-2-one	quercetin-*O*-pentose	quinic acid
hexanal	quercetin-*O*-deoxyhexose	riboflavin
2-hexenal	quercetin-*O*-(*O*-galloyl)-deoxyhexose	serine
(Z)-3-hexen-1-ol	shikimic acid	stearic acid
(E)-2-hexen-1-ol	*O*-coumaroyl-di-*O*-galloyl-hexose	succinate
p-xylene	quercetin-*O*-(*O*-galloyl)-pentose	succinic acid
2-heptanone	(*epi*)afzelechin	
(Z)-4-heptenal	quercetin-*O*-(*O*-coumaroyl)-hexose-hexose-hexose	sucrose

2,5-dimethylpyrazine	dimethyl (epi)gallocatechin gallate	sucrose
benzaldehyde	di-*O*-caffeoylquinic acid methyl ester	tagitinin
1-heptanol	(epi)catechin trimethyl ether	tagitinin c
1-octen-3-ol	fatty acid	tagitinin C epoxide
phenol	trihydroxy-12-oleanen-28-oic acid	taurine
2,3-octanedione	trihydroxy-5,12-oleanadien-28-oic acid	theanine
6-methyl-5-hepten-2-one	trihydroxy-12-ursen-28-oic acid	threonine
β-myrcene	betulinic acid	trigoneline
2-pentylfuran	betulonic aldehyde	trimethylamine
(E,Z)-2,4-heptadienal	oleanolic acid	trimethylamine n-oxide
decane	3-oxo-olean-12-en-28-al	tyrosine
octanal	phaeophorbide b	udp-n-acetylglucosammine
(E,E)-2,4-heptadienal	methyl phaeophorbide b	uridine
limonene	phaeophorbide a	valine
2-ethyl-1-hexanol	ethyl phaeophorbide b	α-glucose
benzyl alcohol	methyl phaeophorbide a	α-phellandrene
(E)-3-octen-2-one	diterpene	α-pinene
phenylacetaldehyde	ethyl phaeophorbide a	β-glucose
1,3,6-octatriene, 3,7-dimethyl-, (z)	*β*-amyrin	β-phellandrene
1-ethyl-2-formyl-1h-pyrrole	PG(18:1(9Z)/22:4 (7Z,10Z,13Z,16Z)	γ-aminobutyric acid
5-ethyldihydro-2	PG(18:0/22:6(4Z,7Z,10Z,13Z,16Z,19Z))	tartaric acid
(3H)-furanone	PG(*O*-16:0/20:2(11Z,14Z))	acetoin
(E)-2-octenal	PS(19:0/22:1(11Z))	arginine
2-acetyl-1h-pyrrole	δ-3-carene	pyruvic acid
acetophenone	oxypeucedanin hydrate	syringic acid
(E,E)-3,5-octadien-2-one	2,3-epoxygeranial	butyric acid
linalool oxide 1	rutin	fumaric acid
meta-cresol	isoquercitrin	2-hydroxyisobutyrate
heptanoic acid	4,5-*O*-dicaffeoylquinic acid	ketoleucine
linalool oxide 2	3,5-dicaffeoylquinic acid	ethyl acetoacetate
3,5-octadien-2-one	quercitrin	galactitol
undecane	3,4-dicaffeoylquinic acid	sorbitol
linalool	luteolin	glutamic acid
nonanal	quercetin	isobutyric acid
(E)-6-methyl-3,5-heptadienone	spermidine	propylene glycol
maltol	nepetin	s-sulfocysteine
2-phenylethanol	tagitinin b	shikimic acid
(E)-4,8-dimethylnone-1,3,7-triene	tagitinin a	trigonelin
isophorone	apidenin	mannitol

benzyl nitrile	hispidulin	alpha-glucose
1-nonanol	hamamelonic acid	ethanal
linalool oxide 4	2-methyl-1,2,3,4-butanetetrol	caffeic acid
naphthalene	4-*O*-methyl-D-glucuronic acid	
(Z)-3-hexenyl butyrate	citric acid	
2,6-dimethylocta-3,7-diene-2,6-diol	protocatechuic acid	
methyl salicylate	chlorogenic acid	
dodecane	caffeic acid	
4-vinylphenol	4-*O*-caffeoyl-2-*C*-methyl-D-threonic acid	
pentan-2-one	2-o-methyltagitinin b	
ethyl acetate	tagitinin e	
3-methylbutanal	phosphoserine	
2-methylbutanal	n-acetylphenylalanine	
pentane-2,3-dione	glutamic acid	
4-methylpentan-2-one	hydroxyproline	
ethyl propanoate	urocanic acid	
methyl butanoate	cysteic acid	
methyl pentanoate	nicotinamide ribotide	
2-methyl-butane-1-thiol	guanine	
octane	5-aminoimidazole-4-carboxamide	
methyl 2-methylpentanoate	N-amidino aspartic acid	
ethyl 3-methylbutanoate	methyl acetoacetic acid	
methyl (E)-2-methylbut-2-enoate	glycerol-3-phosphate	
methyl 3-methylpentanoate	cholic acid	
2-methylbutanoic acid	heptose	
ethyl pentanoate	hexose	
methyl hexanoate	phosphoaspartate	
ethyl (E)-2-methylbut-2-enoate	urea	
ethyl 2-methylpentanoate	trigoneline	
ethyl 3-methylpentanoate	taurine	
ethyl hexanoate	sebacic acid	
butyl pentanoate	ribothymidine	
ethyl 2-hydroxy-4-methylpentanoate	phenylacetylglycine	
ethyl 2-hydroxyhexanoate	malic acid	
2-methylbutyl 3-methylbutanoate	carnitine	
1,3-bis(1,1-dimethylethyl)-benzene	acetylcarnitine	
α-copaene	isovalerylglycine	
α-cedrene	hippuric acid	
caryophyllene	galactonic acid	
α-trans-bergamotene	creatine	
humulene	citrate	
β-santalene	ascorbic acid	
β-farnesene	adenosine	
eugenol	4-guanidinobutanoic acid	

octadecanoid acid	2-methyl-3-ketovaleric acid
γ-tocoferol	calyxolane a
trans-anethole	calyxolane b
proximadiol	methyl trans-monocyclo-farnesate
dehydroabietal	cinachylenic acid A
culinaldehyde	methyl 6-methoxy-3,6-peroxyhexa-deca-4,10,12-trienoate
1-(*m*-methoxycinnamoyl)pyrrolidine	plakortide E
(+)-pinanediol	stylisterol A
dill apiol	2-methyl-2-[(3e,7e,11e)-4,8,12,16-tetramethyl-heptadeca-3,7,11,15-tetraenyl]chromen-6-ol
α-cubenene	2-pentaprenylbenzoquinone
valerenol	difurospinosulin
δ-3-carene	sarcotride a
β-phellandrene	sarcotride d
methyl chavicol	TP1 A
aromadendrene	TP2
γ-bisabolene	TP3
methyl anthranilate	TP4
β-phellandrene	dibromoacetic acid
eucalyptol	bromoiodoacetic acid
oxypeucedanin hydrate	tetrachloroacetone
α-terpinene	bromo trichloroacteone
neryl propanoate	dibromo dichloroacetone
paracymene	tribromo chloroacetone
α-thujene	tetrabromoacetone
2,3-epoxygeranial.	tetrabromobutane dione
chloroacetaldehyde	ipurolic acid
bromochloroacetaldehyde	dihydroxypalmitic acid
chlorodibromomethane	hexadecenoic acid
dibromoacetaldehyde	octadecanedioic acid
1,3-dichloroacetone	hydroxymyristic acid
bromoform	hydroxyhexadecanoicacid
1,1,3-trichloro-2-propanone	rinoleic acid
1,1,3,3-tetrachloro-2-propanone	methylene cholesterol sulfate
3-amino-2,5-dichlorobenzoic acid	L-lysopine
carbon tetrabromide	4-*O*-methylgallic acid 3-*O*-sulphate
1,1-dibromo-3-chloropropanone	*O*-6-deoxy-α-L-galactopyranosyl-(1- > 2)-*O*-β-D-galactopyranosyl-(1- > 4)-2-(acetylamino)-1,5-anhydro-2-deoxy-D-arabino-hex-1-enitol
methyl tribromoacetate	4-(2-amino-3-hydroxyphenyl)-2,4-dioxobutanoic acid
1,1-dibromo-3,3-dichloropropanone	L-carnitine
1,1,3-tribromoacetone	7,8-dihydrolycopene
triiodomethane	fertaric acid

1,1,3-tribromo-3-chloropropanone	gamma-Glutamylmethionine
3,3-dibromo-2-methylprop-2-enoic acid	cincassiol B
1,1,3,3-tetrabromoacetone	cirsiliol
benzoic acid-2,2 bromoethyl – methoxy	LysoPA(18:0/0:0)
malic acid	methyl oxindole-3-acetate
cyclopentene	2-keto-glutaramic acid
formic acid ethyl ester	stearidonic acid
acetone	PA (18:1(9Z)/18:1(11Z))
2-methyl-propanal	valtrate
propanoic acid ethyl ester	3-glucosyl-2,3′,4,4′,6-pentahydroxybenzophenone
2-methyl-propanoic acid ethyl ester	adenylsuccinic acid
n-propyl acetate	3-Hexylpyridine
2-butanol	dTDP-D-glucose
butanoic acid ethyl ester	melleolide C
1-propanol	resveratrol
2-methyl-butanoic acid ethyl ester	ganoderic acid Mj
3-methyl-butanoic acid ethyl ester	7-hydroxy-2-methyl-4-oxo-4H-1-benzopyran-5-acetic acid
1,1-diethoxy-3-methyl-butane	
2-methyl-1-propanol	
(*R*)-(-)-2-pentanol	
3-methyl-1-butanol acetate	
pentanoic acid ethyl ester	
1-butanol	
4-methyl-pentanoic acid ethyl ester	
3-methyl-1-butanol	
butanoic acid butyl ester	
hexanoic acid ethyl ester	
4-ethoxy-2-butanone	
1,1,3-triethoxy-propane	
heptanoic acid ethyl ester	
2,6-dimethyl-pyrazine	
(L)-2-hydroxy-propanoic acid ethyl ester	
2-hydroxy-butanoic acid ethyl ester	
trimethyl-pyrazine	
octanoic acid ethyl ester	
acetic acid	
2-furaldehyde diethyl acetal	
furfural	
1-(2-furanyl)-ethanone	
nonanoic acid ethyl ester	
formic acid octyl ester	
2-methyl-propanoic acid	
5-methyl-2-furancarboxaldehyde	

4-oxo-pentanoic acid ethyl ester
butanoic acid
decanoic acid, ethyl ester
3-methyl-butanoic acid
butanedioic acid diethyl ester
1,2-dimethoxy-benzene
pentanoic acid
benzeneacetic acid ethyl ester
acetic acid 2-phenylethyl ester
dodecanoic acid ethyl ester
hexanoic acid
benzenepropanoic acid ethyl ester
2-diethoxymethyl-3-methyl-butan-1-ol
phenylethyl alcohol
tetradecanoic acid ethyl ester
octanoic acid
ethyl 13-methyl-tetradecanoate
9-oxo-nonanoic acid ethyl ester
pentadecanoic acid ethyl ester
(Z)-ethyl pentadec-9-enoate
hexadecanoic acid ethyl ester*
ethyl 9-hexadecenoate
n-hexadecanoic acid
ethyl hydrogen succinate
ethyl oleate
linoleic acid ethyl ester
benzeneacetic acid
(Z,Z,Z)-9,12,15-octadecatrienoic acid ethyl ester
tetradecanoic acid
